# Effects of grain processing methods on the expression of genes involved in volatile fatty acid transport and pH regulation, and keratinization in rumen epithelium of beef cattle

**DOI:** 10.1371/journal.pone.0198963

**Published:** 2018-06-14

**Authors:** Pedro Del Bianco Benedeti, Breno de Castro Silva, Marcos Vinícius Carneiro Pacheco, Nicola Vergara Lopes Serão, Ivan Carvalho Filho, Mariana Mescouto Lopes, Marcos Inácio Marcondes, Hilário Cuquetto Mantovani, Sebastião de Campos Valadares Filho, Edenio Detmann, Marcio de Souza Duarte

**Affiliations:** 1 Department of Animal Science, Universidade Federal de Viçosa, Viçosa, Minas Gerais, Brazil; 2 Department of Animal Science, Universidade do Estado de Santa Catarina, Chapecó, Santa Catarina, Brazil; 3 Department of Animal Science, Iowa State University, Ames, Iowa, United States of America; 4 Department of Microbiology, Universidade Federal de Viçosa, Viçosa, Minas Gerais, Brazil; Huazhong University of Science and Technology, CHINA

## Abstract

Two experiments were carried out to evaluate the effects of corn and sorghum with different processing methods on the expression of genes involved in volatile fatty acids transport and pH regulation, and ruminal keratinization in rumen epithelium of finishing bulls. For Exp. 1, five rumen cannulated Nellore bulls were used in a 5x5 Latin square arrangement, with 14 d for adaptation and 9 d for sample collection. Treatments were: dry ground corn, dry ground sorghum, reconstituted corn, reconstituted sorghum, and control (forage-based diet). Samples of rumen epithelium from ventral sac were excised, rinsed, snap-frozen and stored at -80°C until total RNA isolation and quantitative real-time PCR analysis. In the Exp. 2, 24 Nellore bulls were assigned to a completely randomized design lasting 168 d. Experimental treatments were similar to those at Exp. 1, but without the control treatment. After the experimental period, bulls were slaughtered and rumen epithelium samples were rapidly excised for further histological analysis. Rumen epithelial tissue from animals fed reconstituted corn had lower expression of downregulated-in-adenoma (*P* = 0.03) and Na+/H+ exchanger 2 (trend; *P* = 0.09). The expression of Na+/ H+ exchanger 1 (*P* = 0.10) and putative anion transporter (*P* = 0.06) tended to be lower in rumen epithelium of bulls fed reconstituted grains. Ruminal concentration of valerate was greater for animals fed reconstituted grain (*P* = 0.01). Likewise, animals fed reconstituted corn tended to have greater butyrate ruminal concentration (*P* = 0.08). Keratinized layer thickness did not differ among treatments (*P* > 0.10). Therefore, reconstituted grains (especially corn) decrease the mRNA expression of genes involved in volatile fatty acids transport and pH control in the rumen epithelium.

## Introduction

Corn and sorghum are typically the primary grains used for finishing cattle in South American feedlots [1]. Due to their high costs, these grains are usually processed to enhance the efficiency of digestion, reducing waste and improving livestock profitability. The main objective of grain processing is to increase the energy availability by facilitating access to grain starch for rumen microorganisms or/and intestinal digestion [2, 3]. Although typical grain processing methods such as; dry corn, may enhance digestibility, there are still high starch losses when animals are fed high-grain levels diets [4]. Consequently, more extensive grain processing methods have been studied aiming to enhance digestibility without causing digestive dysfunctions [5, 6]. A feasible alternative to improve the dry ground grain fermentation is its rehydration (above 26% moisture) followed by anaerobic fermentation to form the reconstituted grain [7, 8]. However, although high ruminal fermentation diets can increase microbial yield [9] and energy available to the animal, there is a risk of developing acidotic epithelial damage, thus affecting nutrient absorption.

It has been suggested that the uptake of volatile fatty acids (VFA) through the rumen epithelium may occur by passive diffusion [10], and also by carrier-mediated transporters [11]. Thus, key proteins would be located in the epithelial cells that regulate intracellular pH regulation function by exporting HCO_3_^-^ or H^+^ from cytosol to ruminal lumen [5]. Hence, understanding the VFA transport through the rumen epithelium at a molecular level could offer the potential to help develop improved nutritional management programs for beef cattle.

Previous studies have reported that the expression of genes associated with VFA transport generates proteins that may have synergistic effects with ruminal pH and VFA concentration [12, 13], which may vary according to the extent of feed fermentation. However, it is unclear how grain processing methods impact expression of genes encoding key proteins favoring the control of VFA, H^+^ and HCO_3_^-^ epithelial transport processes. The studies performed herein were designed to test our hypothesis that animals fed moisture reconstituted grains would have greater VFA transport from rumen lumen to epithelium than those fed dry ground grains. Therefore, the objective of this study was to evaluate the expression of genes involved in VFA transport and pH regulation, and keratinization in rumen epithelium of cattle fed corn and sorghum subjected to different processing methods.

## Material and methods

### Ethical approval

All the experiments were carried out at the Universidade Federal de Viçosa, Viçosa, Minas Gerais, Brazil. Care and handling of all experimental animals were conducted under protocols approved by the Institutional Animal Care and Use Committee of the Universidade Federal de Viçosa (protocol numbers 42/2016 and 29/2017).

### Grain processing

Prior to the beginning of the experiment, a total of 12,000 kg of corn and sorghum grain (6,000 kg each) were ground to pass a 3-mm screen sieve (Wiley mill; Thomson Scientific Inc., Philadelphia, PA) and then analyzed for dry matter (DM, method 934.01; [14]. Then, water was added to 3,000 kg of each grain until DM decreased to 640 g/kg as fed. After moisturized, corn and sorghum were separately ensiled in laboratory silos for 90 days to form the reconstituted grain. Therefore, two grains (corn, and sorghum) with two processing methods (dry ground, and reconstituted) were used in this two-experiment study.

### Animal handling, data collection, and sampling

For **Exp. 1**, five Nellore young bulls [260 ± 23 kg of body weight (BW) and 8 ± 1 mo] with ruminal cannulas were kept in a tie stall equipped with water and feed troughs. Prior to the experiment, all bulls were weighed, vaccinated, and dewormed. Then, animals were adapted to the facilities, and management for 30 d. Bulls were assigned into a 5 × 5 Latin square with five treatments and five replicates per treatment. The experimental periods, lasting 23 d each, encompassed 14 d for adaptation [15] and 9 d for sampling. Experimental diets were composed of 28.4% corn silage and 71.6% concentrate (DM basis). Diets were formulated to be isonitrogenous and meet the nutrient requirements of beef cattle recommendations [16]. Experimental treatments consisted of two ingredients, each one submitted to two processing methods (dry ground corn, dry ground sorghum, reconstituted corn, and reconstituted sorghum) in finishing beef diets, plus a control treatment with dry ground corn and lower concentrate inclusion (45:55 forage to concentrate ratio), which have a usual inclusion of grain in Brazilian beef cattle diets. Ingredient and chemical composition of the experimental diets are presented in Table 1.

**Table 1:** Feeds and chemical composition of experimental diets.

Item		Dry ground		Reconstituted	
	Control	Corn	Sorghum	Corn	Sorghum
Feed, g/kg of dry matter					
Corn silage	450,0	284,4	284,4	284,4	284,4
Dry ground corn	442,7	608,3	-	-	-
Reconstituted corn	-	-	-	608,3	-
Dry ground sorghum	-	-	608,3	-	-
Reconstituted sorghum	-	-	-	-	608,3
Soybean meal	67.5	67.5	67.5	67.5	67.5
Vitamin-mineral premix^1^	29.4	29.4	29.4	29.4	29.4
Urea + Ammonium sulfate^2^	10.4	10.4	10.4	10.4	10.4
Composition, g/kg of dry matter					
Dry matter, g/kg as fed	434	541	539	465	468
Organic matter	929	940	940	938	937
Crude protein	127	132	137	132	136
Neutral detergent fiber [17, 18]^3^	279	204	197	193	192
Non-fiber carbohydrates [19]^4^	500	577	593	587	596

^1^ Premix guarantees (per kg of DM): 200–220 g of Ca, 10 mg of Co (Min), 500 mg of Cu (Min), 22 g of S (Min), 333 mg of Fe (Min), 178.41 mg of F (Max), 10 g of P (Min), 25 mg of I (Min), 17 g of Mg (Min), 1500 mg of Mn (Min), 1100 mg of monensin, 100 x 109 CFU of *Saccharomyces cerevisiae* (Min), 6.6 mg of Se (Min), 50 g of Na (Min), 100,000 IU of vitamin A (Min), 13,000 IU of vitamin D3 (Min), 150 IU of vitamin E (Min), and 2,000 mg of Zn (Min).

^2^ Urea + ammonium sulfate in a 9:1 ratio.

^3^ Neutral detergent fiber corrected for residual ash and residual nitrogenous compounds.

^4^ Non-fiber carbohydrates = 100 − [(crude protein–crude protein from urea + urea) + neutral detergent fiber + ether extract + Ash].

Bulls were fed twice daily at 0800 and 1500 h. Feed bunks were evaluated each day to quantify orts and to adjust daily feed allowance to a maximum of 10% of orts. Corn silage and reconstituted grains were analyzed daily for DM (method 934.01; [14] to adjust ingredient proportions in diets. From day 15 to 23, voluntary intake was calculated by the ratio between the amount of diet offered minus orts. From day 15 to 22, ruminal digestibility was determined using a double marker (iNDF + Co-EDTA) technique. From day 20 to 22, omasal digesta was sampled at intervals of 9 h as follows: day 20, sampling at 0800 h and 1700 h; day 21, sampling at 0200 h, 1100 h, and 2000 h; and day 22, sampling at 0500 h, 1400 h, and 2300 h. Samples were frozen at -80°C, freeze-dried for 72 h, and then ground through a 1-mm screen in a Wiley Mill. At the end of the process, a composite sample was prepared for each animal in each sampling period. The ruminal digestibility coefficient was determined using the average intake and the estimated omasum amount of organic matter (OM) [20]. A pH meter device (Well Cow bolus, Roslin, Edinburgh, Scotland) was used to continuously monitoring the ruminal pH during sampling days.

On day 23 of each experimental period, 20 mL of ruminal fluid were sampled from 5 different sites at 0, 4, and 8 h after feeding. Rumen fluid was filtered through triple layer of cheesecloth. Then, 5 mL of a 25% (w/v) of metaphosphoric acid solution was added for latter determination of VFA. On the same day, sample of rumen epithelium from ventral sac was rapidly excised from 10 different points of the ventral sac of each animal. All samples were immediately rinsed with phosphate-buffered saline (PBS; pH = 7.04), pooled together by each animal, and then snap frozen in liquid nitrogen. Finally, samples were stored at -80°C until total RNA isolation.

The **Exp. 2** was carried out concomitantly with the animals assigned into a 5 x 5 Latin square design. A total of 24 Nellore bulls (270 ± 48 kg of BW) were allocated into collective pens (7.0 m^2^ per animal) equipped with an electronic system for monitoring individual feed intake (AF 1000 Master, Intergado Ltd., Contagem, Minas Gerais, Brazil). After a 28-d adaptation period, the experimental treatments were randomly assigned to bulls in a completely randomized design resulting in six animals per treatment. The experimental period was carried out over 140 d. Experimental treatments consisted of four diets containing either corn or sorghum with two processing methods each (dry ground corn, dry ground sorghum, reconstituted corn, and reconstituted sorghum). Feed and diets compositions were the same of the animals kept in the 5 x 5 Latin square design. Bulls were fed twice daily at 0800 and 1500 h, allowing for up to 10% orts. After the experimental period, bulls were harvested at the experimental abattoir of the *Universidade Federal de Viçosa*. Pre-harvest handling was conducted in accordance with good animal welfare practices, and harvesting procedures followed strict guidelines established and regulated by the Sanitary and Industrial Inspection Regulation for Animal Origin Products [21]. Animals were stunned using a pneumatic penetration pistol and then immediately bled. After harvest, a sample of rumen epithelium (10 cm^2^) from ventral sac was rapidly excised from each animal, washed with phosphate-buffered saline (PBS, pH 7.04), and then fixed in formalin (10% v/v) for 24 h. Finally, formalin-fixed tissues were immersed in ethanol (70% v/v) and stored for further histological analysis.

### Volatile fatty acids (VFA) molar ratio in rumen fluid

Rumen fluid samples (2.0 mL) were thawed at room temperature, centrifuged at 12,000 x g for 10 min. Then, cell-free supernatants were treated as described by Siegfried et al. [22]. Volatile fatty acid concentrations were quantified using a Dionex Ultimate 3000 Dual detector HPLC (Dionex Corporation, Sunnyvale, CA, USA) coupled to a refractive index (RI) Shodex RI-101 maintained at 40°C. The ion exchange column (300 x 7.8) used was a Phenomenex Rezex ROA (Phenomenex Inc. Torrance, CA, USA), which was maintained at 45°C. Mobile phase was prepared with 5 mmol/L H_2_SO_4_, and the flow was set as 0.7 mL/min. The following volatile fatty acids were used to calibrate the standard curve: acetic, succinic, formic, lactic, propionic, valeric, isovaleric, isobutyric and butyric acid. The volatile fatty acids were prepared with a final concentration of 10 mmol/L, except isovaleric acid (5 mmol/L) and acetic acid (20 mmol/L).

### Total RNA extraction and mRNA expression in rumen epithelium tissue

Total RNA extraction was performed from 50 mg of rumen epithelium samples using RNeasy Mini Kit (Qiagen, Valencia, CA). After that, total RNA was subsequently treated with DNAse I Amplification Grade (Invitrogen, Waltham, MA) and RNA concentration was estimated by NanoVue Plus spectrophotometer (GE Healthcare, Freiburg, Germany). The RNA integrity was evaluated through agarose gel electrophoresis. The first strand of cDNA synthesis was performed using a GoScript Reverse Transcriptase kit (Promega, Madison, WI, USA). Samples were stored at -20°C for further analysis. The VFA transport associated genes used in this study were: *anion exchanger 2* (*AE2*), *downregulated-in-adenoma* (*DRA*), *monocarboxylic acid transporter 1 and 4* (*MCT1*, *MCT4*), *Na*^*+*^*/ H*^*+*^ *exchanger 1*, *2*, *and 3* (*NHE1*, *NHE2*, and *NHE3*), *putative anion transporter* (*PAT1*), and *vacuolar-type ATPase* (*H*^*+*^*ATPase*). Primers for target gene amplification and endogenous amplification were designed using PrimerQuest program (www.idtdna.com/Scitools/Applications/PrimerQuest) with sequences obtained from GenBank database. Primer sequences of each gene are presented in Table 2. The *18S ribosomal RNA* (*18S*; NR_036642.1) was used as the endogenous control gene. Serial dilution of cDNA was used to determinate the amplification efficiency and optimal primer concentration for each gene [23]. Quantitative real-time PCR reactions were performed in an ABI Prism 7300 Sequence Detection Systems thermocycler (Applied Biosystems, Foster City, CA, USA) using a GoTaq qPCR Master Mix (Promega Corporation, Madison, WI, USA). The qPCR reaction consisted of three cycle parameters: 95°C for 3 min, 40 cycles at 95°C for 10 s, and 60°C for 30 s. The amplification efficiency was 0.90 to 0.99. After amplification, a melting curve (0.01°C/s) was used to confirm product purity. Results are expressed relative to 18S using the ΔCt method [23]. No variation of 18S expression was observed (*P* > 0.05) among experimental treatments. Target cycles (Ct) values greater than 35 cycles were considered undetectable expression.

**Table 2:** PCR primers for detection of target populations.

Gene	Abbreviation	Primer sequences (5'-3')	NCBI^1^ accession No.	Amplicon Size
Anion exchanger 2	*AE2*	F: AGCAGCAACCTGGAGT	NM_001205664.1	122
		R: GGTGAAACGGGAGACGAA		
Downregulated-in-adenoma	*DRA*	F: TTTAAAGTCCTAGAGTCCGTA	NM_001083676.1	112
		R: CGCTGATTTATTTCTTTAACCAC		
Monocarboxylic acid transporter 1	*MCT1*	F: ACCAGTTTTAGGTCGTCTCA	NM_001037319.1	106
		R: GGCTTCTCAGCAACATCTACA		
Monocarboxylic acid transporter 4	*MCT4*	F: GTTTGGGATAGGCTACAGTGACACA	NM_001109980.1	105
		R: GCAGCCAAAGCGATTCACA		
Na^+^/ H^+^ exchanger 1	*NHE1*	F: CCTCTACAGCTACATGGCCTAC	NM_174833.2	112
		R: GGGAGATGTTGGCTTCCA		
Na^+^/ H^+^ exchanger 2	*NHE2*	F: TTGGAGAGTCCCCTGCTGAAC	NM_003048.3	140
		R: GGCCGTGATGTAGGACAAAT		
Na^+^/ H^+^ exchanger 3	*NHE3*	F: AGCTACGTGGCCGAGGG	NM_004174.2	145
		R: AGACAGAGGCCTCCAACGGT		
Putative anion transporter	*PAT1*	F: CCTTGAGGCACGGCTAC	NM_078483.2	125
		R: GCACCAGACTCCGAGACATA		
Vacuolar-type ATPase	*H*^*+*^ *ATPase*	F: TTTTATTGAACAAGAAGCCAATGA	NM_175796.2	115
		R: GATTCATCAAATTGGACATCTGAA		

^1^ NCBI, National Center for Biotechnology Information database (www.ncbi.nlm.nih.gov).

### Rumen epithelium keratinization

Samples of rumen epithelium were collected from cattle that were slaughtered at the end of the feeding trial. Samples were fixed in fresh 10% (wt/vol) formalin in phosphate buffer (pH 7.4). Next, tissue samples were embedded in Paraplast (Sigma-Aldrich, St. Louis, MO) and cut into sections of 5 μm by using a RM2245 microtome (Leica Microsystems Inc.). Sections were rehydrated through incubations on Histochoice (Sigma-Aldrich) and ethanol solutions. Following the rehydration, sections were stained with eosin and hematoxylin, and mounted in synthetic resin. Mounted slides were observed under light microscopy for evaluation of rumen epithelium keratinization by using an EVOS xl light microscope (AMG, Bothell, WA). The thickness of keratinized epithelium was measured at several points on each papillae from the base up to the apical area. Measurements from each papillae were averaged on each image and a total of 50 images per animal were evaluated. Images were taken at 200-fold magnification by using ImageJ software (National Institute of Health, Baltimore, MD).

### Statistical analysis

Data from animals assigned into a 5 x 5 Latin square design were analyzed using the following model:

$$Y_{ijk}=\mu +{Diet}_{i}+p_{j}+a_{k}+e_{ijk}$$

where, *Y*_*ijk*_ is the observed measurement; *μ* is the overall mean; *Diet*_*i*_ is the fixed-effect of the *i*^*th*^ level of dietary treatment (5 levels); *p*_*j*_ is the random effect of the *j*^*th*^ level of period (5 levels), with *p*_j_ ~ *N*(0, *σ*_p_^2^); *a*_*k*_ is the random effect of the *k*^*th*^ animal (5 levels), with a_k_ ~ *N*(0, *σ*_a_^2^); and *e*_*ijk*_ is the random error associated with *Y*_*ijk*_, with e_ijk_ ~ *N*(0, *σ*_e_^2^). A series of orthogonal contrasts were constructed in order to test biologically relevant questions for the fixed-effect of *Diet*. The first contrast tested the difference between the Control diet and the average of all other 4 diets. After that, the other 3 remaining orthogonal contrasts were used to test the effects of Ingredient, Processing method, and their interaction. Prior to the final analyses, the residuals from the analysis of each trait were assessed for normality using Shapiro-Wilk’s test. As expected, the gene expression data was not normal and it was transformed using *ln*(2^-ΔCt^ + 1) [24]. Least-squares means were estimated for the effect of Diet, Ingredient, Processing method, or their interaction when needed.

Values of rumen epithelium keratinization obtained from animals that were assigned into a completely randomized design, were analyzed in a model that included the fixed effects of Diet (4 levels). Least-squares means were separated using Fisher’s least significant difference test. Results were deemed significant when *P* ≤ 0.05 and trending when 0.05 < *P* ≤ 0.10. All analyses were performed with the GLIMMIX procedure in SAS 9.4 (Statistical Analysis System Institute, Inc., Cary, NC, USA).

## Results

No differences (*P* > 0.10) were observed for OM intake in g/kg of BW, which averaged 17.3 ± 3.1 g/kg of BW (Table 3). However, animals fed dry ground grains had greater OM intake in kg/day (trend; *P* = 0.06), rumen degraded OM (RDOM; *P* < 0.01), and ruminal digestibility of OM (*P* = 0.03) than animals fed reconstituted grains. Digestibility variables were also affected by ingredients; sorghum treatments tended (*P* = 0.08) to have higher OM ruminal digestibility than corn. Control treatment showed lower OM ruminal digestibility (*P* = 0.03), compared to remain treatments.

**Table 3:** Effect of ingredients and processing methods on organic matter (OM) intake and digestibility in Nellore bulls.

Item^1^		Dry ground		Reconstituted		SEM	*P*-value			
	Control	Corn	Sorghum	Corn	Sorghum		Control x Remaining treatments	Ingredient	Processing method	Ingredient x processing method
OM Intake										
kg/day	5.33	5.69	5.38	4.68	5.25	0.62	0.80	0.63	0.06	0.14
g/kg of BW	17.5	18.3	17.6	15.7	17.5	0.95	0.79	0.57	0.19	0.21
RDOM, kg/day	2.63	3.20	3.22	2.41	2.87	0.37	0.11	0.14	<0.01	0.18
OM ruminal digestibility, g/kg	499	565	591	505	554	21.8	0.03	0.08	0.03	0.57

^1^ SEM, standard error of the mean; OM, organic matter; BW, body weight; RDOM, rumen degraded organic matter.

No differences were observed for rumen concentration of acetate (*P* > 0.10), which averaged 12.5 ± 3.3 mmol/L (Table 4). Control treatment tended to have lower concentrations of valerate (*P* = 0.10) and *iso*-butyrate (*P* = 0.09), compared to other treatments average. Only valerate concentration was affected by grain processing methods, being greater (*P* = 0.01) for bulls fed reconstituted grains. Bulls fed sorghum had greater acetate: propionate ratio in rumen fluid than those fed corn (*P* = 0.01). On the other hand, bulls fed corn had greater concentrations of total VFA (*P* = 0.03), propionate (*P* < 0.01), valerate (*P* < 0.01), *iso*-butyrate (*P* = 0.04), and *iso*-valerate (*P* = 0.01) in rumen fluid than those fed sorghum. Moreover, reconstituted corn tended to have greater butyrate (*P* = 0.08), and *iso*-valerate (*P* = 0.07) concentrations, compared with other treatments.

**Table 4:** Effect of ingredients and processing methods on pH, and total and individual volatile fatty acids (VFA), in rumen fluid of Nellore bulls.

Item^1^		Dry ground		Reconstituted		SEM	*P*-value			
	Control	Corn	Sorghum	Corn	Sorghum		Control x Remaining treatments	Ingredient	Processing method	Ingredient x processing method
pH	6.29	5.97	6.12	5.92	6.13	0.17	0.07	0.13	0.86	0.81
Total VFA, mmol/L^2^	18.4	19.8	18.6	24.5	18.6	2.34	0.25	0.03	0.13	0.96
VFA profile, mmol/L^2^										
Acetate	11.9	12.3	12.0	14.7	11.8	1.54	0.46	0.12	0.26	0.19
Propionate	3.57	4.30	3.34	5.13	3.67	0.51	0.18	<0.01	0.11	0.47
Butyrate	1.95	2.01	2.18	2.83	2.03	0.29	0.29	0.23	0.20	0.08
Valerate	0.15	0.19	0.14	0.31	0.18	0.31	0.10	<0.01	0.01	0.16
*Iso*-butyrate	0.26	0.32	0.28	0.40	0.29	0.04	0.09	0.04	0.16	0.26
*Iso*-valerate	0.46	0.59	0.49	1.01	0.46	0.13	0.19	0.01	0.11	0.07
Acetate: propionate	3.40	2.86	3.55	2.99	3.22	0.18	0.13	<0.01	0.49	0.13

^1^ SEM, standard error of the mean; VFA, volatile fatty acids.

^2^ Values are corrected for kg of organic matter intake.

Rumen epithelium from animals fed reconstituted corn had lower mRNA expression of *DRA* (Interaction ingredient x grain processing; *P* = 0.03) and tended to have lower mRNA expression of *NHE2* (*P* = 0.09), compared with other treatments (Table 5). The mRNA expression of *NHE1* (*P* = 0.10) and *PAT1* (*P* = 0.06) tended to be lower in rumen epithelium of bulls fed reconstituted grains than dry ground grains. The mRNA expression of *AE2*, *MCT1*, *MCT4*, *NHE1*, and *NHE3* did not differ among treatments.

**Table 5:** Effect of ingredients and processing methods on relative mRNA expressions of genes involved in volatile fatty acids transport in rumen epithelium of Nellore bulls.

Item^1^		Dry ground		Reconstituted		SEM^2^	*P*-value			
	Control	Corn	Sorghum	Corn	Sorghum		Control x Remaining treatments	Ingredient	Processing method	Ingredient x processing method
AE2	3.36	3.67	3.17	2.84	3.07	0.76	0.82	0.83	0.49	0.59
DRA	2.70^a^	3.02^a^	2.99^a^	1.80^b^	3.17^a^	0.55	0.90	0.04	0.10	0.03
MCT1	1.79	1.97	1.77	1.56	1.62	0.36	0.83	0.78	0.27	0.61
MCT4	1.59	1.82	2.03	1.72	1.95	0.31	0.38	0.46	0.75	0.97
NHE1	1.58	1.84	1.74	1.22	1.68	0.25	0.86	0.39	0.10	0.19
NHE2	2.05	2.24	2.05	1.50	2.07	0.42	0.72	0.36	0.10	0.09
NHE3	1.76	1.63	2.00	1.46	1.78	0.28	0.89	0.23	0.48	0.92
PAT1	3.07	3.25	3.25	2.58	3.17	0.34	0.95	0.13	0.06	0.12
H^+^ ATPase	3.89	4.03	3.30	2.90	3.53	0.65	0.43	0.93	0.38	0.19

^1^ ΔCt values of relative expression to 18S

^2^ SEM, standard error of the mean; AE2, *anion exchanger 2*; DRA, *downregulated in adenoma*; MCT1, *monocarboxylate transporter 1*; MCT4, *monocarboxylate transporter 4*; NHE1, *Na*^*+*^*/H*^*+*^ *exchanger 1*; NHE2, *Na*^*+*^*/H*^*+*^ *exchanger 2*; NHE3, *Na*^*+*^*/H*^*+*^ *exchanger 3*; PAT1, *putative anion transporter*; H^+^ATPase, *vacuolar-type ATPase*.

^a, b^ Within a row, different subscripts differ at *P* < 0.05.

**Fig 1:**
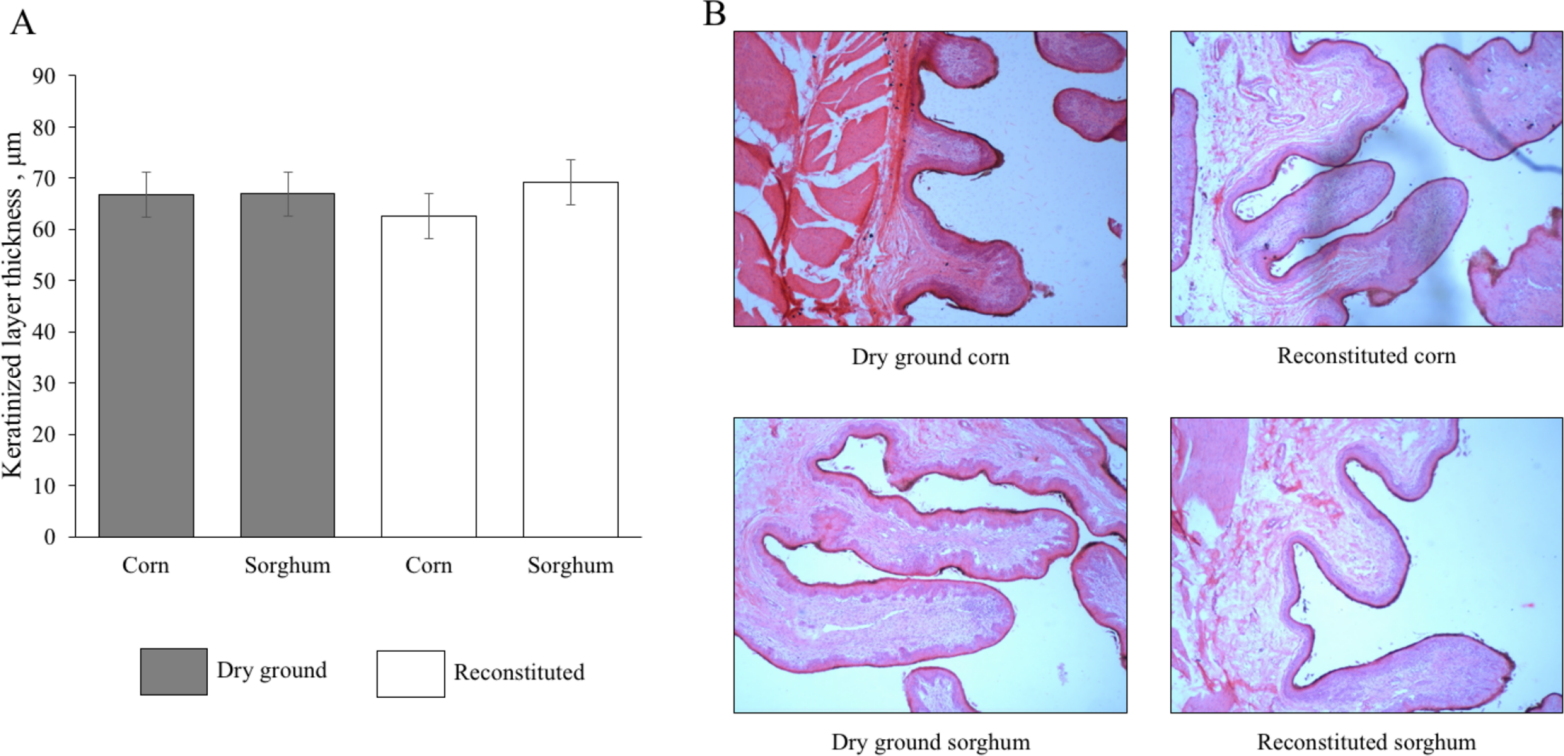
A—Effect of ingredients and processing methods on keratinization in rumen epithelium of Nellore bulls. B—Longitudinal section from rumen tissue of Nellore bulls fed dry ground corn, dry ground sorghum, reconstituted corn, and reconstituted sorghum, showing the normal histology of these animals. Standard error of the mean, 4.37; Ingredient *P*-value, 0.45; Processing method *P*-value, 0.83; Ingredient x Processing method *P*-value, 0.47 (Fisher’s least significant difference test).

No differences (*P* > 0.10) were found for keratinization of rumen epithelium of Nellore bulls fed dry ground corn, dry ground sorghum, reconstituted corn, and reconstituted sorghum (Fig 1).

## Discussion

Different carbohydrate sources as well as different grain processing may lead to changes in VFA production, as it might change the ruminal fermentation pattern. Consequently, the absorption of VFA will depend on the pH of rumen content, fluid flow to abomasum, and the capacity of the rumen epithelium to adapt to the rumen environment. Here we hypothesized that the animals fed reconstituted grains would present greater mRNA expression of key genes involved in VFA transport in their ruminal epithelium than animals fed dry ground grains. Thus, we decided to evaluate potential differences in mRNA expression of *AE2*, *DRA*, *MCT1*, *MCT4*, *NHE1*, *NHE2*, *NHE3*, *PAT1*, and *H*^*+*^*ATPase*, which are known as markers for regulation of VFA, H^+^ protons and HCO_3_^-^ epithelial transport processes [5, 25, 26].

**Fig 2:**
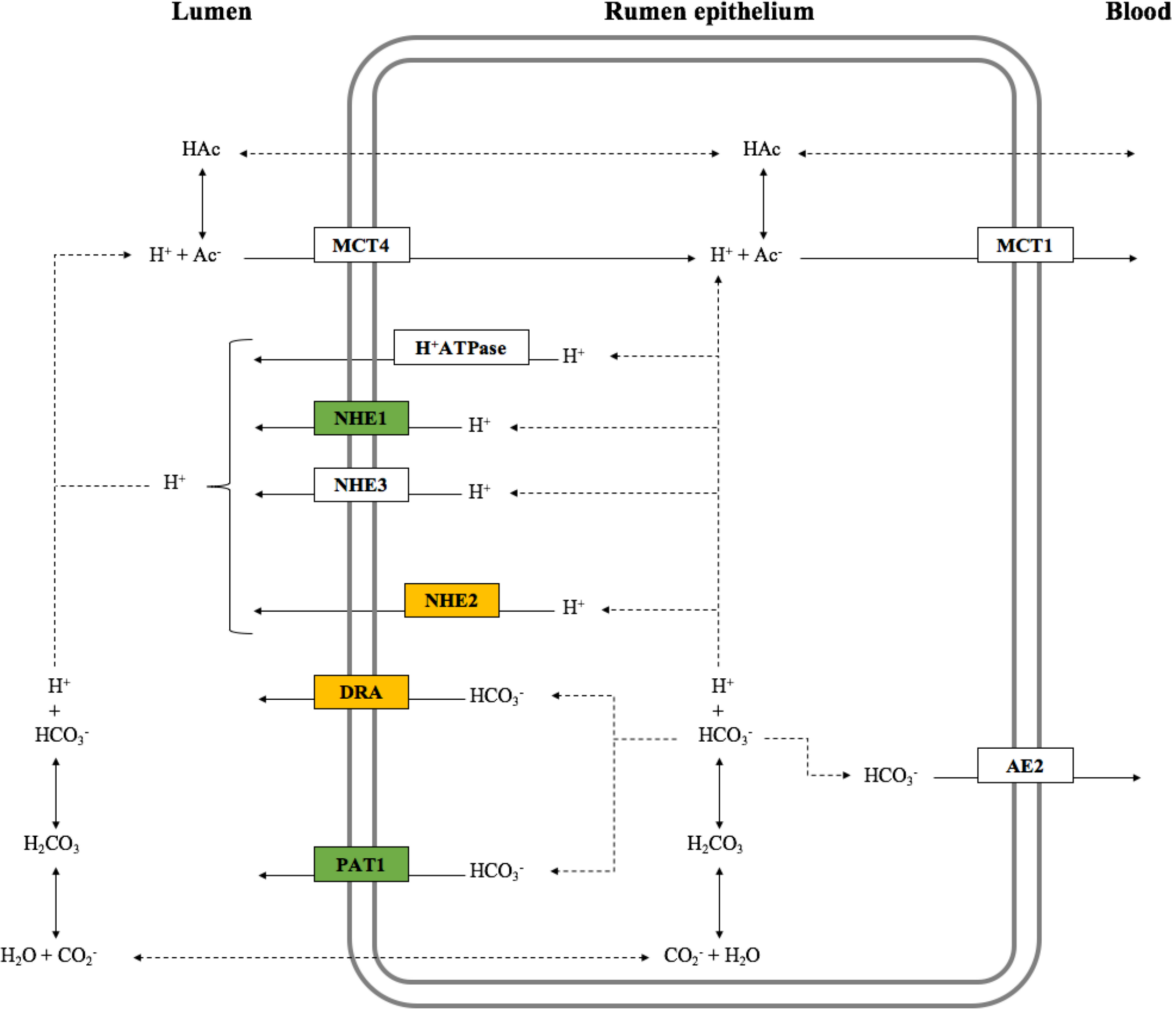
Mechanisms of volatile fatty acid transport through rumen and proteins involved in pH control of rumen epithelium [Adapted from Stevens and Hume [30] and Connor et al. [5]]. Genes with lower expression in the rumen epithelium of animals fed reconstituted corn are presented in orange [*DRA* (*P* = 0.03), and NHE2 (trend; *P* = 0.09)]. Genes that tended to have lower mRNA expression in the rumen epithelium of animals fed reconstituted grains are presented in green [*NHE1* (*P* = 0.10), and *PAT1* (*P* = 0.06)]. AE2 = *anion exchanger 2*; DRA = *downregulated in adenoma*; MCT1 = *monocarboxylate transporter 1*; MCT4 = *monocarboxylate transporter 4*; NHE1 = *Na*^*+*^*/H*^*+*^ *exchanger 1*; NHE2 = *Na*^*+*^*/H*^*+*^ *exchanger 2*; NHE3 = *Na*^*+*^*/H*^*+*^ *exchanger 3*; PAT1 = *putative anion transporter*; H^+^ATPase = *vacuolar-type ATPase*.

Contradicting our hypothesis, animals fed reconstituted grains tended to have lower mRNA expression of *NHE1*, and *PAT1* in their rumen epithelium than those fed dry ground grains. Furthermore, animals fed reconstituted corn had lower mRNA expression of *NHE2* (Trend), and *DRA*, compared to remaining treatments. These genes have opposite functions in pH control of the ruminal lumen and epithelial cells (Fig 2). The *DRA* and *PAT1* main function in the rumen epithelium is transport HCO_3_^-^ from inside the cell to the ruminal lumen [26]. This favors the pathway of absorption of non-ionized VFA into cytosol, facilitating its uptake by the blood. However, the higher HCO_3_^-^ concentration in the lumen increases the proportion of ionized VFA in the rumen, which may decrease VFA transport into the rumen epithelium cells by passive diffusion. On the other hand, the major role of *Na*^*+*^*/H*^*+*^ *exchanger family* (*NHE*) is to regulate the intracellular pH of the rumen epithelium by translocating H^+^ from cytosol to the lumen, counteracting its high concentration caused by the VFA transport into the rumen epithelium cell [11, 27]. Previous studies have demonstrated that factors such as fermentation products and pH may work as modulators of gene transcription of rumen epithelium VFA transporters [28, 29]. Therefore, the expression of genes with opposite functions (e.g. NHE2 and DRA for reconstituted corn) on lumen and cytosol pH control, may be regulated to maintain a constant pH in rumen epithelium, contributing for VFA transport.

Because mechanisms underlying feed intake and digestibility may impact VFA production and absorption through rumen epithelium of finishing bulls, we have assessed the OM intake and ruminal digestibility of animals fed corn and sorghum with different processing methods (dry ground and reconstituted). Once our digestibility results suggest that there is a greater feed digestion in post-rumen compartments of the gastrointestinal tract (especially small and large intestines), the underlying mechanisms regarding nutrients absorption by these other tissues should be the focus of further investigation. With regards to the control versus other treatments evaluation, the greater pH and lower OM digestibility observed for control are in accordance with the more presences of forage in this diet and fiber indigestible components in corn silage.

Among VFA, butyrate and valerate are those with greater role in papillae development in the rumen [31, 32]. Furthermore, VFA absorption might be related with ruminal papillae surface area, which would allow greater VFA diffusion transport through rumen tissue. Moreover, variations in ruminal butyrate concentration may have influence on blood flow rate [33], which is also related with VFA uptake [34, 35]. Therefore, despite the lower mRNA expression of genes involved in pH control for reconstituted grain (especially corn), butyrate (greater for reconstituted corn) and valerate (greater for reconstituted grain) results suggest that VFA transport is more related with passive diffusion processes through rumen tissue of animals fed this treatment. As observed here, Laarman et al. [36] found an increased molar proportion of butyrate but decreased expression of *Na*^*+*^*/H*^*+*^ *exchanger* (subunit 3; *NHE*3) for calves fed calf starter compared with control treatment. However, these authors also observed that the former treatment had greater mRNA expression of MCT1, which is a co-transporter that contributes to proton transport from ruminal epithelium to blood. These authors suggested that the greater MCT1 expression could be occurred to complement the lower expression of NHE3 on net proton transference from rumen epithelium cells. Nevertheless, mRNA expression of MCT1 did not differ among treatments in our study.

Given the proven effect of grain processing on ruminal fermentation enhancement [8], the possible occurrence rumen mucosal damage is a concern when rapid rumen fermentation is expected. As rumen epithelium acts as a protective barrier between the rumen environment and the portal circulation, rapid fermentation patterns may lead to an increase of keratinized layers of the rumen epithelium [37]. Keratinization of rumen epithelium which has been associated with decreased absorption capacity of nutrients, as a consequence of the metabolically active tissue reduction [38], which may lead to a decrease in animal performance during the finishing period. In the present study, we have hypothesized that animals fed reconstituted grains have greater rumen fermentation than animals fed dry ground grains and thus stimulate the development of rumen papillae. Contradicting our hypothesis, the thickness of keratinizatized layer of the rumen epithelium did not differ (*P* > 0.05) among treatments. Therefore, our results suggest that the grain processing methods evaluated does not cause any deleterious effect on rumen epithelium that would compromise the ruminal absorption processes.

## Conclusion

Reconstituted grains can decrease the OM ruminal digestibility and mRNA expression of genes involved in VFA transport and pH control in the rumen epithelium. From a physiological perspective, these results indicate that the expression of pH regulators, with complex functions, may be regulated to maintain a constant pH in rumen epithelium, contributing for VFA uptake.

## Supporting information

S1 Table Raw data. (XLSX)

## Abbreviations

AE: anion exchanger 2
BW: body weight
DM: dry matter
DRA: downregulated-in-adenoma
H+ATPase: vacuolar-type ATPase
iNDF: indigestible neutral detergent fiber
MCT1, MCT4: monocarboxylic acid transporter 1, and 4
NHE1, NHE2, NHE3: Na^+^/ H^+^ exchanger 1, 2, and 3
OM: organic matter
PAT1: putative anion transporter
RDOM: rumen degraded organic matter
VFA: volatile fatty acids
